# Adoption of innovative and evidence-based practices for children and adolescents in state-supported mental health clinics: a qualitative study

**DOI:** 10.1186/s12961-017-0190-z

**Published:** 2017-03-29

**Authors:** Lawrence A. Palinkas, Mee Young Um, Chung Hyeon Jeong, Ka Ho Brian Chor, Serene Olin, Sarah M. Horwitz, Kimberly E. Hoagwood

**Affiliations:** 1 Department of Children, Youth and Families, Suzanne Dworak-Peck School of Social Work, University of Southern California, 669 W. 34th Street, Los Angeles, CA 90089-0411 United States of America; 2 Chapin Hall at the University of Chicago, Chicago, IL United States of America; 3 Department of Child and Adolescent Psychiatry, New York University School of Medicine, New York, United States of America

**Keywords:** Innovation, Adoption, Evidence-based treatments and practices, Mixed methods, Child and adolescent mental health

## Abstract

**Background:**

This study examined how mental health clinic administrators decided whether or not to adopt evidence-based and other innovative practices by exploring their views of implementation barriers and facilitators and operation of these views in assessment of implementation costs and benefits.

**Methods:**

Semi-structured interviews were conducted with 75 agency chief executive officers and program directors of 34 New York State-licensed mental health clinics serving children and adolescents.

**Results:**

Three interconnected themes relating to barriers and facilitators were identified, namely costs and benefits associated with adoption, capacity for adoption, and acceptability of new practices. The highest percentage of participants (86.7%) mentioned costs as a barrier, followed by limited capacity (55.9%) and lack of acceptability (52.9%). The highest percentage (82.3%) of participants identified available capacity as a facilitator, followed by acceptability (41.2%) and benefits or limited costs (24.0%). Assessment of costs and benefits exhibited several principles of behavioural economics, including loss aversion, temporal discounting use of heuristics, sensitivity to monetary incentives, decision fatigue, framing, and environmental influences.

**Conclusions:**

The results point to opportunities for using agency leader models to develop strategies to facilitate implementation of evidence-based and innovative practices for children and adolescents.

## Background

Despite substantial evidence of their effectiveness, evidence-based practices (EBPs) in mental health services for children and adolescents continue to be underutilised [1, 2]. In the past decade, much research has been devoted to understanding the reasons for the underutilisation of EBPs in all forms of mental health services and how this situation can best be remedied so that clients can receive the highest quality of care [3–5]. This research has led to a proliferation of EBP implementation models that identify characteristics of the EBP itself, the organisations and individuals tasked with implementing the intervention, and the external environment in which such implementation occurs, which may either serve as implementation barriers or facilitators [6–8]. The development of strategies to facilitate EBP implementation has relied upon this work to identify the barriers that must be overcome and the facilitators that can assist in this task.

Although there seems to be general consensus as to the potential of such theories, models and frameworks for facilitating implementation of EBPs and other innovative practices, some [9, 10] have questioned their value for understanding and guiding implementation and pointed to their limitations for connecting implementation theory with practice. For instance, frameworks and models in general provide checklists of potential determinants of successful implementation but do not prioritise them or indicate which are most important or predictive of implementation outcomes and which are the least [11]. Further, as Proctor et al. ([12], p. 72) observe, “*the success of efforts to implement evidence-based treatment may rest on their congruence with the preferences and priorities of those who shape, deliver, and participate in care. Implementation outcomes may be differentially salient to various stakeholders, just as the salience of clinical outcomes varies across stakeholders*”. A study by Seffrin et al. [13] found that the proportion of facilitators to the sum of facilitator and barrier comments made by project informants were higher for innovative mental health projects that proceeded with implementation than those that did not.

Related to the lack of information on stakeholder preferences and priorities for implementation is a limited understanding of the principles and processes of deciding whether or not to adopt, implement and sustain a new and innovative practice. Panzano and Roth [14] found that the propensity to adopt an innovative mental health practice was negatively related to the perceived risk of adopting the practice and positively related to expected capacity to manage risk and an organisation’s past propensity to take risks. Whether leaders of mental health service agencies consider factors in addition to risk aversion when making decisions on whether or not to adopt an innovative program or practice is not entirely clear.

To better understand the key factors involved in deciding whether or not to adopt an EBP or other innovative practice, we conducted a qualitative study to construct an agency leadership model of implementation by exploring the following: (1) the most salient or important barriers and facilitators to innovation and adoption of EBPs for children and adolescents as determined by executive directors and program directors of mental health clinics in New York State; and (2) how these barriers and facilitators operate in the process of weighing the costs and benefits of implementation.

## Methods

### Participants

The present study included 75 agency CEOs, vice presidents and program directors representing a 10% random stratified sample of 346 clinics (*n* = 34) licensed by the New York State Office of Mental Health (OMH) to treat youths in New York State. These 34 clinics were randomly selected based on their level of adoption of EBPs in New York State, as operationalised in Chor et al. [15]. After completing informed consent procedures for participating in research, each participant completed a semi-structured interview conducted between August 2013 and June 2014. The number of interviewees per clinic ranged from one to three. The study was reviewed and approved by the Institutional Review Boards at New York University and the University of Southern California.

### Data collection

All interviews were conducted using a semi-structured interview guide consisting of a series of questions that focused on understanding (1) why OMH-licensed, outpatient, child-serving agencies and clinics adopt innovations in the context of healthcare reform; and (2) multi-level (system-, agency-/clinic-, staff-, client- and innovation-levels) processes and factors in adoption, non-adoption and de-adoption of innovations. Participants were informed at the outset that innovations can be EBPs for specific psychiatric disorders; they can also be quality improvement initiatives, which are activities that improve the structures, processes and outcomes of care of a clinic. Questions were drawn from previous studies of EBP dissemination and implementation [16, 17] and were designed to obtain information on knowledge of innovations (e.g. How does your agency generally first hear about an innovation?), innovations adopted (e.g. What types of innovations has your agency adopted?), adoption decision-making (e.g. What motivates or drives your agency to adopt an innovation?), and facilitators and barriers of adoption of innovations. In this last category of questions, participants were asked the following: (1) What are the major factors (facilitators) that make it possible to adopt these innovations? (2) What are the major factors (barriers) that make it difficult to adopt these innovations? and (3) What are the top three barriers? All interviews lasted approximately 1 hour and were digitally recorded.

### Data analysis

A thematic content analysis methodology of “Coding Consensus, Co-occurrence, and Comparison” [18] was used to analyse the semi-structured interviews. Audio-recorded interviews were transcribed and reviewed by four investigators, who developed lists of codes individually. These codes were subsequently discussed, matched and then integrated into a single codebook. The final list of codes, constructed through a consensus of team members, consisted of responses to specific questions related to perceived barriers and facilitators to adoption of innovative practices. Inter-rater reliability in the assignment of specific codes to specific transcript segments was assessed for a subset of randomly selected pages from 10 randomly selected transcripts. For all coded text statements, the coders agreed on the codes 91% (range, 88–94%) of the time, indicating good reliability in qualitative research [19]. A web-based qualitative data management program, Dedoose [20], was used for coding and generating a series of categories arranged in a treelike structure connecting text segments as separate categories of codes or ‘nodes’. Through repeated comparisons of these categories with one another, these nodes and trees were used to create a taxonomy of themes that included both a priori and emergent categories and new, previously unrecognised categories. These nodes and trees were used to further the process of axial and pattern coding [21].

As every interview participant was asked to identify the three top barriers and facilitators, we quantified the number of responses to these answers by category or theme and subcategory to determine what barriers and facilitators were most often mentioned as an indicator of their significance or salience.

## Results

A list of barriers and facilitators to adopting innovative and EBPs and the percentage of study participants who mentioned a specific barrier or facilitator is presented in Table 1 below. The highest percentage of participants (86.7%) mentioned costs as a barrier, followed by limited capacity (55.9%) and lack of acceptability (52.9%). With respect to facilitators, the highest percentage (82.3%) of participants identified available capacity, followed by acceptability (41.2%) and benefits or limited costs (24.0%).

**Table 1:** Percentage of study participants identifying specific adoption barriers and facilitators

Barriers	Percent	Facilitators	Percent
Costs	86.7	Benefits or limited costs	24.0
Time for training	54.4	Available time	19.4
Training expenses	47.1	Evidence of positive outcomes	14.9
Impact on organisation	23.5	EBP flexibility	7.4
No evidence of outcomes	8.8	Little impact on organisation	6.0
Staff turnover	5.9	Free/low costs	6.0
EBP requirements	4.4		
Barriers		Facilitators	
Capacity	55.9	Available capacity	82.3
Lack of trained staff	19.1	Training access	44.7
Organisational resources	17.6	Financial support and incentives	40.3
Environmental constraints	13.2	Available trained staff	38.8
Financial reimbursement	8.8	Support from leadership	16.4
Training access	7.4	Organisational resources	14.7
Lack of technical support	2.9	Regulatory mandate	11.9
Lack of leadership support	1.5	Supervision/consultation	11.9
		Presence of a champion	4.5
		More information	3.0
		Interagency network	1.5
Acceptability	52.9	Acceptability	41.2
Staff fit and buy-in	32.4	Staff motivation to change	14.9
Client fit and buy-in	23.5	Organisational fit and leadership support	29.8
Organisational fit and leadership support	14.7	Client need	14.9
		Other agencies implementing	1.5

### Costs and benefits of adoption

Analysis of the qualitative data revealed three central themes of barriers and facilitators to implementing EBPs. The first theme focused on issues of cost and included additional time required to train staff, direct costs, reduced productivity, staff turnover, resource requirements for use of the EBP, and lack of evidence of the innovation’s benefits. Parallel to the theme of costs as a barrier were benefits and reduced costs as a facilitator. This theme included available time, evidence of positive outcomes associated with the innovation, flexibility in use of the innovation, little impact on the organisation and availability of the innovation for free or at low cost.

The cost of implementing a new program or practice was the most frequently cited barrier. One agency chief executive officer explained, “*…the first thing I look at is how much is this gonna cost us if we try to implement it. Because I mean, right now finances are a mess through most of the system for agencies that are working with kids, especially the size of* [our agency], *that’s a small to medium agency*”.

The most frequently cited cost was associated with the training of staff. Such training involves two types of costs, the cost of the training itself and the lost revenues that occur when staff members are not seeing clients while they are getting trained. Training costs include the expense of paying for someone skilled in the practice to train one’s staff, purchasing of treatment manuals or other instructional aids, and perhaps travel expenses of staff who are sent elsewhere for training. Most importantly, training results in lost productivity and revenue “*because as soon as you’re sending someone to a training, they’re not doing billable services*” (Program director). This, in turn, results in reduced productivity of the agency as a whole. On the other hand, the availability of training for free or low cost was cited as a facilitator to adoption: “*Yeah, so certainly any organised external effort or webinar or the ability to send people to a training on an evidence based practice that is comprehensive and free, is one that we…that certainly makes it easier for us to participate in*” (Agency CEO).

In addition to lost revenues and productivity, participants cited other costs associated with the impact of adoption on the organisation as a whole. A number of participants cited the additional paperwork associated with the adoption of a new practice. Others cited the additional workload that would affect staff morale because it would make it more difficult “*to be able to stay current, to read, to take a deep breath, to look at things…*” (Agency CEO).

Lack of evidence of positive outcomes is another barrier because it calls into question the benefits of adopting the innovation. As one agency CEO observed, “*I’m not going to take a bunch of clients who are doing fairly well and switch them over to some other treatment mode, unless I have a fairly good sense that they’re going to do as well, or if not a little bit better. And I don’t always get that all right*”.

Another cost associated with training is potential staff turnover. As explained by one program director: “*You’re investing in a staff member for, let’s say a year in a training program and then they think that experience is great, and they go to another place… People don’t see how much it costs to the agency, unfortunately*”.

Related to the cost of training staff are the costs involved in supervising staff to use the practice with fidelity: “*Because now if you have any new model, somebody has to oversee it and supervise it. And that again, becomes a money and time factor of who’s going to have the time to now supervise this model and make sure it’s being done effectively*” (Program director). Practicing with fidelity also may require additional time, which can reduce productivity: “*So for example, the CPT requires forty-five minutes. That means that for the clinicians that are implementing this model, we don’t have that flexibility of scheduling their patients in those thirty-minute slots to increase productivity*” (Agency vice president). In contrast, flexibility in adapting the innovation to the practice was considered a facilitator to adoption.

### Capacity for adoption

The second theme focused on issues of clinic capacity to implement the EBPs. Barriers included lack of trained staff, lack of organisational capacity and resources, environmental constraints, lack of financial reimbursement for implementing the innovation, limited access to training, lack of technical support, and lack of support from leadership. Facilitators related to capacity for adoption included available training, funding or financial support and incentives, trained staff, leadership that was supportive of the adoption, organisational resources, a regulatory mandate, supervision, the presence of an innovation champion, more information about the innovation, and access to an interagency network.

While the additional costs associated with training and supervising staff and an increased workload and time devoted to using the innovation with fidelity may be viewed as a disincentive to adoption, there is the additional risk of not being adequately reimbursed for incurring these costs. One of the program directors cited a specific practice, dialectical behaviour therapy, as an illustration: “*I think it’s very hard to implement in an outpatient mental health clinic. Maybe it won’t be, once we have those capitated rate, but the way that things are now where you do a fee per session model doesn’t really work with DBT* [dialectical behaviour therapy]”. An agency vice president also noted: “*you can’t bill for the type of supervision that’s required to remain with the fidelity of the model*”. Consequently, “*one of the major barriers to provision of services to children, is they are highly labor intensive and not reimbursed at such wonderful levels as it’s easy to afford them*”.

Even if one were to obtain reimbursement for training and supervision, the limited access to useful resources is also an important barrier to adoption. Participants noted the paucity of high quality training opportunities and the absence of supervision as a follow-up to training as barriers to adopting innovations. According to one agency CEO, “*We’ve been trained in a number of these practices. But there is not a depth in New York State to provide fidelity based supervision*”.

Lack of trained staff is another important barrier. This includes the lack of staff qualified to use the innovation, the lack of staff available to be trained, and the lack of support staff. As one program director noted: “*Yes. I think the number one* [barrier] *is just not having enough staff. And that’s really the main thing because not enough staff everybody takes on more tasks. You know, it seems like we’re always asking people to do more. The staff here haven’t had raises in four years*”.

Another barrier to adoption relates to constraints on capacity imposed on an agency by its external environment. Often mentioned by participants was the challenge of providing mental health services to school-age clients during school hours. Another environmental constraint is the absence of a regulatory policy governing implementation. This constraint is also associated with the limited capacity to obtain reimbursement for using the innovation. For instance, one agency vice president noted a challenge with the transition to Medicaid managed care: “*We’ve had problems with managed care companies approving evidence based models for group therapy because they don’t want to pay for it, which then you’ve got to fight them, appeal it, you know that kind of stuff. So I think it’s a lot of those things that really are the barriers*”.

However, regulations that mandate services may also be seen as a facilitator in that they eliminate the need to make a decision: “*Yeah, the most obvious is if there’s a regulatory change I have no choice. I mean, I get to go to my boss and say, we have no choice we have to do this in order to be compliant*” (Agency CEO).

Related to the lack of fit is a lack of support from the agency’s leadership. As noted by one program director, “*barriers are definitely if the executive team doesn’t agree with what we’re trying to do or doesn’t feel that it is the appropriate initiative to expend a lot of time and energy on*”.

The presence of a champion for the innovative program or practice within the agency was cited as a facilitator that enhanced an agency’s capacity to adopt an innovation. While innovation champions could be senior administrators or supervisors, they may also be frontline staff. In either case, “*you’ve got to have some people who are excited about it, who take it on and who believe, and who are able to communicate enthusiasm to the other staff and help support them through the implementation… If you don’t have that then you can’t change the culture*” (Program director).

Another facilitator related to capacity is the ability to provide financial incentives to staff. As explained by an agency director, “*So… a clinician reaches one hundred percent productivity, they are full time staff in the union, salary, benefits, they reach a hundred percent and then they go over a hundred percent. They’re fabulous; they have a great week. We would like to incentivize that and say for every session you go over we can either reimburse you at a fee for service rate, we can give you a gift card for twenty-five dollars, you know, something*”.

Providing incentives to clients was also considered a facilitator, “*Well, you know, our clients, some agencies give our clients to come in, they give them gifts and food. We don’t have incentives here. Clients have been incentivized. And we provide no incentives, but they come in. So sometimes an agency around the corner will be giving out, just to give you an example, will be giving a fifty dollar gift card to come in. They’ll go there, get the fifty dollar gift card but then they eventually come back*” (Agency CEO).

Other distinct facilitators reflecting the capacity to adopt included prior experience with adopting other innovations, the existence of a quality improvement unit within the agency, being in a supportive academic environment, and having more information about the innovation itself.

### Acceptability of adoption

The third theme focused on the acceptability of the EBPs from the perspective of the staff responsible for using them, the agency as a whole, and the clients or consumers who are the presumed beneficiaries of the EBPs. Staff resistance was the most frequently cited barrier, “*When you’re working with evidence-based practices that are different from what people are used to doing, you certainly have to overcome staff resistance*” (Agency vice president).

Lack of fit with the client and the client’s needs and preferences is another barrier. The fit with client needs is usually expressed in terms of a positive outcome. Lack of a fit between the practice requirements and the client’s preference is considered a barrier: “*It’s also the patient resistance to change and the lack of cooperation from the parents*” (Program director).

Finally, lack of fit of the innovation with the organisation is another barrier, as one clinic manager noted, “*And obviously, yeah, anything that would go against the agency’s mission, clearly, you know that we’re not going to touch*”.

Facilitators related to acceptability of the innovation included staff motivation to change current practices, evidence of positive outcomes in addressing client need and preferences, and the existence of a supportive leadership and organisational culture. For instance, related to evidence of positive outcomes was the manner in which the information related to the innovation was presented or ‘framed’. As explained by one program director, “*What motivates? Well, just how it’s presented to me, how I present it to the staff meetings. Is it presented to the managers the same way, basically saying this is the new program, for instance, that we’re going to be using and how this will be effective for clients? So I like to see examples how it may help another program. Or I like to see numbers and see how effective it is…. So once I see those numbers, then I’m driving to make sure that it’s in my program*”.

The only facilitator related to acceptability that was not the opposite of staff buy-in, organisational fit, and client need and buy-in was the observation that other agencies were also adopting the innovation. As expressed by one agency CEO, “*So if the health system’s doing it, then obviously it’s easy for us to do. If other hospitals are doing it and we want to compete with other hospitals, or sometimes we need to show that we’re doing it too*”. A desire to remain in good standing with OMH was also viewed as a facilitator in that it created a pressure to adopt the innovation. This pressure was cited by one program director: “*OMH, you know is pushing for person recovery…center recovery, so that’s what we’re looking at. You know, we’re trying to integrate that into our treatment approach*”.

### Weighing costs and benefits

Although participants were asked to identify barriers and facilitators separately, it was evident in several instances that both factored into some form of assessment or calculation of the benefits relative to the costs. There were several references to being motivated by EBPs as being ‘cost effective’ and the need to determine whether “*the clinical value of the program or even the efficiency value is an investment that we can afford to do, basically. We need to make that choice*” (Agency CEO). Benefits were viewed both in terms of improved outcomes for the individual client as well as improved productivity or revenues for the agency as a whole. In some instances, this assessment is made with the recognition that the benefits are not immediate, but are likely to occur at some point in the future. In other instances, the benefits are not entirely clear, “*Because some of the trainings we pulled people out of their billing time. So we make sacrifices. And I don’t know if it’s worth it, but we do…*” (Program director).

However, two particular patterns were observed in the assessment of costs and benefits. The first pattern was a focus almost entirely on the costs of adoption with an expressed desire to avoid these costs with little or no attention given to benefits. In many instances, the costs are perceived to clearly outweigh the benefits. For instance, one program director expressed an unwillingness to adopt “*…interventions that just don’t fit…that are not user friendly, that are hard to fit into our practice, that don’t seem to fit with the population that we’re serving. Interventions that are very time consuming and require a lot of other documentation or require a lot of additional time. And then interventions that just don’t seem like they’re effective. They just don’t make sense*.”

In another instance, unless the innovative practice is short-term, it may not be cost-effective because “*the longer you’re working with a family the more questions and challenges there are around the effectiveness of our work*” (Agency CEO).

The second pattern was the reliance on untested assumptions in assessing costs and benefits. For instance, references to staff resistance to change in general were often based on personal experience and did not appear to rely on ‘data’ based on the specific instance (i.e. a particular innovation), “*You know, like certain…I know, within myself and sense that maybe with colleagues, there’s a certain resistance to change*” (Program director).

## Discussion

Our analyses of the perceived barriers and facilitators of innovation adoption revealed an agency leadership model of implementation based on assessments of the cost and potential benefits of adoption, the organisation’s capacity to adopt, and the acceptability of adoption to the client, providers and the organisation itself. The cost of adopting was the most frequently cited barrier, followed by the organisation’s capacity to adopt and the acceptability/fit of the innovation to clients, staff and the organisation. In contrast, the available capacity for adoption was the most frequently cited facilitator of adoption, followed by acceptability and limited costs and potential benefits.

In many respects, the agency leadership model prioritises the salient factors in existing models and frameworks of adoption and implementation developed by researchers [6–8]. For instance, if we take the Consolidated Framework for Implementation Research [7] as an illustration, all of the barriers and facilitators identified by study participants reflect characteristics of innovation (e.g. costs associated with implementing the program, need for and availability of training and supervision evidence of positive outcomes, flexibility, adaptability and perceived excellence in how the program is presented), the external environment or outer setting/context (availability of funds, client needs, regulatory mandates, pressure from OMH and other agencies), the inner setting/context (organisational structure, availability of trained staff, fit with organisational culture, leadership support, financial incentives for implementation), characteristics of individuals (staff fit and buy-in), or implementation process (presence of program champions). Costs, capacity (fidelity) and acceptability also represent three of eight implementation outcomes in the model proposed by Proctor et al. [12]. Elements of the Consolidated Framework for Implementation Research and other implementation frameworks that are not especially salient in the agency leadership model include the role of interorganisational networks, ability to test programs on a small scale, client advocacy, planning, and evaluation or fidelity monitoring.

The model also exhibits five of the six characteristics of the innovation as described in Roger’s Diffusion of Innovations Theory [22]. Relative advantage is represented in the benefit of improved clinical outcomes. Compatibility is represented in the barriers and facilitators related to organisational fit and leadership support and staff buy-in and motivation to change. Complexity is represented in EBP requirements and flexibility. Observability is represented as improved clinical outcomes and client acceptability. Trialability was not represented in participant comments.

The agency leadership model of innovation adoption revealed in this study also illustrates several principles of behavioural economics [23–27]. In contrast to traditional economic theory, which assumes that individuals make rational decisions in order to maximise the utility or personal benefits derived from their choices, behavioural economics recognises the systematic biases inherent in decision making and the notion of ‘bounded rationality’ [27], and posits that utility is a subjective concept representing the individual’s personal satisfaction with the decision [24]. The principles of behavioural economics were summarised by Rice [26] as follows, (1) people tend to be overly oriented to the present rather than the future (temporal discounting); they are more concerned about losing something they have than about gaining something they have not yet experienced (loss aversion) and are very sensitive to monetary incentives, especially those that are most tangible; (2) people are cognitively limited, using heuristics or rules of thumbs to make complex decisions rather than going through all possible choices, they exhibit decision fatigue, which accounts for a preference for less rather than more choice, and they are influenced by how choices are framed (framing); and (3) people’s preferences are influenced greatly by the environment and can be manipulated, especially through advertising.

In this study, clinic leaders do appear to compare the costs and benefits of adoption in accordance with principles of classical economic theory and models of comparative effectiveness research [28]. Benefits include improved outcomes that address the needs of clients and expanded service capacity. However, consistent with the principle of loss aversion, the leaders also tend to focus more on the costs than they do on the benefits of innovation adoption. Costs were identified by the largest percentage of study participants (85.2% versus 14.9% who perceived positive outcomes as a benefit). They also expressed concern over loss of staff that received additional training, and reduced staff morale. Examples of the use of heuristics included the untested assumption that staff would resist adoption in general or that it would require too much time to implement the innovative practice. In these instances, there was no evidence that leaders had polled their staff to determine how receptive they would be to a particular innovation and no evidence of a willingness to experiment (i.e. adopt the innovation on a trial basis). Consistent with the principle of temporal discounting, some agency administrators expressed concern over whether the long-term benefits in clinic efficiency and client outcomes were worth the short-term costs associated with lost productivity during training. Consistent with the principle of framing, agency administrators may be motivated to adopt based on how the innovation is presented to them and how they, in turn, present it to their staff. Consistent with the principle of incentives, participants made reference to the incentive of having staff trained for free or at low cost and incentivising use of the innovation by both clients and practitioners as a potential facilitator to adoption, one that none of the agencies studied appeared to possess at present. Consistent with the principle of decision fatigue and preference for limited choices, participants expressed a preference for mandates as a facilitator because it eliminated the need to make a decision. Reliance on mandates and the pressure to adopt to remain in good standing with the New York State Office of Mental Health to motivate adoption is also consistent with the principle of preferences being influenced by the environment that can be manipulated, along with other agencies’ adoption of innovation, as motivation to adopt.

However, for the individuals making the decision to innovate, the determinants of successful implementation can be reduced to answers to three simple questions, (1) What will it cost me to innovate? (2) Do I have the capacity to innovate? and (3) Am I motivated to innovate? As the results indicate, the same information may be used to answer more than one question. Thus, having a supportive leadership reflects both a capacity for innovation and a desire to do so from an organisational perspective. Evidence of positive outcomes associated with the innovation may be viewed as a benefit contrasted with the costs as well as a motivation by staff, clients and organisations for adopting the innovation.

More importantly, the agency leadership model presented here suggests that the barriers and facilitators are prioritised such that decision-makers give greater weight to costs than to capacity or acceptability when evaluating barriers, but give greater weight to capacity than to costs and acceptability when evaluating facilitators. The most important costs from the perspective of agency decision-makers are temporal and monetary. The lack of trained staff is the most significant or salient barrier related to capacity. The reluctance of staff to adopt new practices is the most significant or salient barrier related to acceptability. Availability of time to innovate and evidence of positive outcomes associated with the innovation are the most important facilitators. Access to training activities and materials and availability of financial support and incentives are the most important capacity facilitators. Organisational fit and leadership support are the most important acceptance facilitators. Thus, while existing implementation frameworks, theories and models may help to guide implementation practice by identifying potential barriers and facilitators that might be important to address when undertaking an implementation endeavour, they are more like checklists of factors relevant to various aspects of implementation [11]. Decision-making models such as the ones used by agency leaders in mental health clinics in New York State are potentially useful in identifying the most important factors within a specific context or setting and devoting limited resources and strategies to addressing these factors.

Finally, the data from this study seem to suggest key factors that researchers should pay attention to and test in quantitative models. In an earlier study with the entire cohort of mental health clinics in New York State from which this sample was drawn, Olin et al. [29] found clinic size, efficiency and outsourcing were significantly associated with participation in business trainings and proportion of youth clients and full-time clinical staff were associated with participation in clinical trainings. The present study enriches those findings by highlighting the potential importance of local/contextual factors not available in state administrative data sets. This knowledge should be used in combination with state administrative data to help states develop targeted strategies for promoting innovation adoption and implementation.

This study has several limitations. As a qualitative investigation, the generalisability of these findings is limited to a sample of senior administrators of mental health clinics serving children and adolescents in New York State. The specific needs and perspectives of this stakeholder group on barriers and facilitators to adopt will likely differ from those of other stakeholders, including state officials, therapists, non-clinical staff and clients. Surveys of a random sample of each stakeholder group would increase the generalisability of these results. Further, the reliance on answers provided to specific questions during the interviews may limit the identification and quantification of barriers and facilitators that may be found in other parts of the interview. Although findings were consistent with theories of behavioural economics, the questions were not specifically designed to examine adoption from that point of view and, therefore, information about tradeoffs, etc., may have been missed. Finally, the cross-sectional design of the study also limits our ability to establish a causal linkage between perceptions of barriers and facilitators and degree of engagement in innovation adoption and implementation. Related to this, the findings of this study relate primarily to the early or initial stages of implementation and may not include perceptions of barriers and facilitators associated with sustainment of such innovations.

## Conclusion

Despite these limitations, our findings illustrate an agency leadership model of innovation adoption reflecting certain principles of behavioural economics. There appears to be a high level of consensus as to perceived costs and benefits, capacity to adopt and acceptability of the innovation. Furthermore, the findings point to areas where current models can be enhanced or modified, particularly with respect to the prioritisation of barriers and facilitators, as well as to the need to take local stakeholders into consideration when developing strategies to facilitate implementation of EBP and innovative practices for children and adolescents.
